# Global Brain Functional Network Connectivity in Infants With Prenatal Opioid Exposure

**DOI:** 10.3389/fped.2022.847037

**Published:** 2022-03-14

**Authors:** Rupa Radhakrishnan, Ramana V. Vishnubhotla, Yi Zhao, Jingwen Yan, Bing He, Nicole Steinhardt, David M. Haas, Gregory M. Sokol, Senthilkumar Sadhasivam

**Affiliations:** ^1^Department of Radiology and Imaging Sciences, Indiana University School of Medicine, Indianapolis, IN, United States; ^2^Department of Biostatistics and Health Data Science, Indiana University School of Medicine, Indianapolis, IN, United States; ^3^Indiana University School of Medicine, Indianapolis, IN, United States; ^4^Department of Obstetrics and Gynecology, Indiana University School of Medicine, Indianapolis, IN, United States; ^5^Department of Pediatrics, Indiana University School of Medicine, Indianapolis, IN, United States; ^6^Department of Anesthesia, Indiana University School of Medicine, Indianapolis, IN, United States; ^7^Department of Anesthesiology and Perioperative Medicine, University of Pittsburgh Medical Center, University of Pittsburgh School of Medicine, Pittsburgh, PA, United States

**Keywords:** prenatal opioid exposure, resting state brain networks, neonatal opioid withdrawal syndrome, rs-fMRI, opioid use disorder

## Abstract

**Background:**

Infants with prenatal opioid and substance exposure are at higher risk of poor neurobehavioral outcomes in later childhood. Early brain imaging in infancy has the potential to identify early brain developmental alterations that may help predict behavioral outcomes in these children. In this study, using resting-state functional MRI in early infancy, we aim to identify differences in global brain network connectivity in infants with prenatal opioid and substance exposure compared to healthy control infants.

**Methods and Materials:**

In this prospective study, we recruited 23 infants with prenatal opioid exposure and 29 healthy opioid naïve infants. All subjects underwent brain resting-state functional MRI before 3 months postmenstrual age. Covariate Assisted Principal (CAP) regression was performed to identify brain networks within which functional connectivity was associated with opioid exposure after adjusting for sex and gestational age. Associations of these significant networks with maternal comorbidities were also evaluated. Additionally, graph network metrics were assessed in these CAP networks.

**Results:**

There were four CAP network components that were significantly different between the opioid exposed and healthy control infants. Two of these four networks were associated with maternal psychological factors. Intra-network graph metrics, namely average flow coefficient, clustering coefficient and transitivity were also significantly different in opioid exposed infants compared to healthy controls.

**Conclusion:**

Prenatal opioid exposure is associated with alterations in global brain functional networks compared to non-opioid exposed infants, with intra-network alterations in graph network modeling. These network alterations were also associated with maternal comorbidity, especially mental health. Large-scale longitudinal studies can help in understanding the clinical implications of these early brain functional network alterations in infants with prenatal opioid exposure.

## Introduction

Opioid use and misuse is an ongoing public health crisis. Opioid use disorder (OUD) in pregnant women in the U.S. has increased considerably over the last few decades (1, 2). Opioid related morbidity and mortality were initially driven by the abuse of prescription opioids in the 1990s (3) followed by marked rise in heroin use around 2010 (4), and later by increasing use of synthetic opioids such as illicitly manufactured fentanyl starting in 2013 (5). Multiple opioids may be misused simultaneously with or without other non-opioid substances (6). According to the Centers for Disease Control and Prevention 2019 data, 7% of women reported using prescription opioids during pregnancy, of whom more than one in five reported opioid misuse (1). The number of opioid involved deaths has doubled in pregnant women from 2007 to 2016 (7). In pregnant women with untreated OUD, fluctuating opioid levels in the maternal bloodstream and periods of withdrawal lead to fetal distress, negatively impacting fetal development and increasing the odds of still birth, poor fetal growth and preterm birth (8–12). Infants with prenatal opioid exposure (POE) are at a high risk for developing withdrawal symptoms after birth–neonatal opioid withdrawal syndrome (NOWS)–which occurs in 55–94% of these infants, and results in prolonged and expensive hospital stays (12–17). Children with a history of NOWS are also at risk for adverse long-term developmental outcomes that may persist to school age, including poor cognitive scores, impaired executive functioning and worse fine motor scores (18–21).

There are a small handful of neuroanatomical studies published so far that have evaluated POE in the fetus, neonate, and infant. These studies have suggested alterations in regional brain volumes (22, 23), microstructure (24–26), and impaired brain functional connectivity (24, 27, 28). However, these aforementioned studies did not specifically assess for the potential effects of maternal comorbidities. Pregnant women with OUD have a high prevalence of coexisting comorbidities including psychological illnesses such as depression, stress and anxiety (29–33), polysubstance use (34), and smoking (35). A few neuroimaging studies have attempted to assesses infant brain development in the presence of these maternal high risk factors individually, with or without polysubstance exposure, but there needs to be a more comprehensive understanding the role of these common concomitant maternal comorbidities in the setting of POE, specifically, their potential influence fetal and infant brain development (35–37).

The human brain grows rapidly in the fetal period and during infancy. Identifying alterations in normal brain developmental trajectory is beneficial in understanding brain function in developmental disorders (38–40). Graph-theoretical network modeling has previously been used to understand normal brain development and has revealed highly efficient topological architectures in the early life (41). Early brain structural connectome network topologies in the infant are shown to have predictive ability for later life cognition and behavior (41–44). Here, we applied brain functional network modeling techniques to understand global brain network properties in infants with POE. The aim of our study was to identify differences in global brain network connectivity in infants with prenatal opioid and drug exposure compared to healthy control infants, using resting-state functional MRI performed at <3 months completed postmenstrual age. We also assessed for associations of maternal comorbidities such as smoking, polysubstance use and maternal psychological factors on infant brain global network connectivity.

## Methods

### Subject Recruitment

This is a prospective study performed at Indiana University (IU) Health with approval by the Indiana University Institutional Review Board. In this study we recruited infants with POE as well as control infants without prenatal opioid exposure at <3 months corrected postmenstrual age. Written informed consent was obtained from at least one parent for all minor participants. All infants enrolled in the study were delivered at IU Health Methodist hospital. Recruitment of pregnant women in the opioid exposed group occurred primarily through the antenatal opioid use disorder clinic at IU health, where buprenorphine is prescribed for medication assisted treatment. The antenatal opioid treatment clinic, where pregnant women with OUD were evaluated by maternal addiction specialists for individualized treatment also had a maternal psychiatrist to assess for and manage any psychiatric or psychological illnesses in these women with OUD. Additionally, we recruited women with OUD who received perinatal care at the IU health clinics and were either on methadone maintenance program for opioid used disorder or were not on a formal opioid maintenance program. Opioid use disorder was documented by urine toxicology positive for opioids antenatally and/or at delivery. Antenatal and perinatal health records were reviewed to obtain details of maternal opioid replacement therapy medications (type and dose). In addition, detailed maternal self-report questionnaires were used to assess presence and severity of maternal smoking and details of polysubstance use (other illicit opioids, non-opioid substances). A documented clinical diagnosis of depression, generalized anxiety disorder, or post-traumatic stress disorder during pregnancy was considered a concomitant maternal psychological factor.

Infants with major genetic or congenital anomalies, or significant postnatal abnormalities such as birth asphyxia or neonatal sepsis were excluded. Medical records were reviewed to collect information regarding maternal and infant demographics, infant birth, and postnatal details including any treatment for NOWS. Our institution has a standardized management plan for infants with POE, which includes inpatient monitoring for at least 5 days. Withdrawal symptoms are scored using the modified Finnegan scoring system every 4 h to decide on need for first-line medication therapy with morphine. For this study, we used the maximum recorded modified Finnegan score, length of postnatal hospital stay, and need for postnatal opioid therapy to control NOWS as individual metrics for assessing clinical severity of NOWS.

### Magnetic Resonance Data Acquisition

All MR imaging on infants was performed on a single research-dedicated 3 Tesla Siemens Prisma (Erlangen, Germany) using a 64-channel head coil. Brain MRI was performed during natural sleep with feed and swaddle technique using a vacuum swaddle device. Earplugs and earmuffs (Natus minimuffs) were used for ear protection. Structural imaging was performed that included high resolution 3D T1-weighted MPGRAGE sequence at 1 x 1 x 1 mm resolution (TR = 2010 ms, TE = 2.91 ms, TI = 1,610 ms, flip angle= 12°, 192 mm field-of-view, 120 slices, and GRAPPA acceleration iPAT = 2) and high-resolution axial T2-weighted imaging with a Turbo-Spin-Echo pulse sequence at high in-plane resolution (0.9 × 0.9 mm, 1 mm slice thickness, TR = 9550 ms, TE = 145 ms). Blood oxygen level dependent (BOLD) contrast resting-state functional MRI (rs-fMRI) was performed using a single-shot EPI sequence with a multiband factor of 3, TR = 1,205 ms, TE = 30.4 ms, 480 volumes with an isotropic resolution of 2.5 mm, and 9:45 min acquisition time.

### MR Processing

Preprocessing of the MR images for each subject was performed using the FMRIB (for Functional MRI of the Brain) software Library (FSL, Oxford, UK) (45). Skull stripping and removal of non-brain tissue was performed on the T2 weighted anatomic images using brain segmentation with the FSL BET tool (46). The first 10 volumes of from the rs-fMRI BOLD were excluded to allow for magnetic equilibration. Fieldmap and gradient-non-linearity distortion corrections were performed using FSL-topup (47). Head motion parameters (volume-to-reference transform matrices, and corresponding rotation and translation parameters) of the rfMRI datasets were estimated and were used for realignment of BOLD volumes using linear motion correction through FSL-MCFLIRT (24). Second order motion derivatives, namely frame-wise displacement (FD) and Derivative of root mean square VARiance over voxelS (DVARS) were also obtained to threshold BOLD data in subsequent CAP analysis (additional details provided in Supplement 1) (48–50). CSF and white matter signal was regressed from the functional volumes. Global intensity normalization was performed across the time series. For each subject, a multi-step approach was used to register the realigned rfMRI BOLD volumes to first, the individual subject T2W anatomic image and next, to the UNC neonatal brain template space using Advanced Normalization Tools (ANTs) (25, 26). 90 regions of interest (ROIs) from the UNC segmented neonatal atlas was then warped to the individual subject BOLD images to extract individual ROI time course data (51, 52). This produced individual subject level mean time course in the ROIs that was used for subsequent analysis.

### CAP Regression

The Covariate Assisted Principal (CAP) regression (53) is a recently developed method to identify brain subnetworks. We used this model to assess differences in brain functional connectivity between infants with and without POE. After standardizing the BOLD signals, whole-brain functional connectivity was obtained by the covariance matrix of the time courses. The CAP method identifies a linear projection of the covariance matrices that captures the individual variations corresponding to the covariate of interest. In the regression model, sex, and gestational age were included to adjust for confounding. For inference, 95% confidence intervals were constructed using 500 bootstrap samples after identifying the linear projections. The method was implemented using the R package cap available on CRAN. This method identified groups of connected ROIs that formed networks that were significantly different between the infants with and without POE. We assessed separately, the association of maternal comorbidities and NOWS severity with these CAP networks, and for each network, used the Bonferroni method to correct for multiple comparisons.

### Graph Network Analysis

In these networks identified by CAP analyses, we applied graph network analysis to extract network characteristics. Initial step for graph network analysis includes binarization of the brain functional network. This was necessary, since correlations between temporal fMRI signal are range −1 to 1, but several of the network measures on graph analyses are designed for binary graphs, i.e., whether connections are either present or not. Therefore, we first applied a sparsification procedure to binarize all the functional brain connectivity networks (54). Instead of using one arbitrary correlation as cutoff, we carefully selected a percentage threshold which can lead to more stable results (55). We then binarized the functional brain network using different percentage threshold (from 10 to 98%) and identified the number of nodes in the largest connected components. Each significant CAP component comprised a set weight for all 90 ROIs. We used threshold 0.15 to identify a subset of ROIs with greatest difference in network connectivity between the opioid exposed and control infants (additional details provided in Supplement 1). The subnetwork involving these ROIs was extracted and binarized. These subnetworks were examined with seven network-level topological measures including assortativity coefficient, flow coefficient, density, global efficiency, modularity, clustering coefficient and transitivity (56). Finally, general linear model in R was applied to evaluate the difference of each network measure between two groups of subnetworks. For each subnetwork, FDR correction was used to correct for the seven network-level topological measures analyzed. Details of graph network-level topological measurements are provided in Supplement 1.

## Results

### Demographics

We recruited 23 infants (8 male) with POE and 29 healthy control infants (15 male) without prenatal opioid exposure. Eighteen of the mothers of infants with POE were on buprenorphine therapy in pregnancy (2–20 mg per day), four were on methadone, and one was not on any opioid maintenance in pregnancy. Polysubstance use included opioid substances such as heroin (4), fentanyl (3) hydrocodone (1) and oxycodone (1), not-specified (2), and non-opioid substances such as methamphetamine (1), marijuana (2), cocaine (2), cannabis (2), and benzodiazepines (2). A significantly larger proportion of women with OUD in pregnancy were smokers and had associated clinically diagnosed depression, generalized anxiety disorder or post-traumatic stress disorder. Demographic details are provided in Table 1. On MRI, review of anatomic images revealed no major anatomical brain abnormalities in either group.

**Table 1:** Demographics of study population.

	**Opioid-exposed (*n* = 23)**	**Controls (*n* = 29)**	***p*** **-value**
Male, *n* (%)	8 (35)	15(52)	0.22
Gestational age at birth (weeks), mean (SD)	37.87 (2.64)	39.23 (0.76)	0.02
Birth weight (Kg), mean (SD)	2.73 (0.52)	3.32 (0.37)	0.52
APGAR score 1 min, mean (SD)	8.39 (0.94)	7.93 (1.67)	<0.001
APGAR score 5 min, mean (SD)	8.83 (0.49)	8.76 (0.74)	0.03
Head circumference at birth (cm), mean (SD)	33.01 (2.33)	34.35 (1.65)	0.22
Postmenstrual age at scan (weeks), mean (SD)	44.13 (3.15)	44.64 (2.18)	0.70
**Infant race/ethnicity**			
Non-hispanic white	20	18	0.57
Non-hispanic black	1	10	0.07
Hispanic white	1	1	0.57
Non-hispanic mixed	1	0	n/a
Maternal depression/stress/anxiety, *n* (%)	12 (52)	4 (14)	0.03
Maternal smoking, *n* (%)	15 (65.2)	0	n/a
Any maternal alcohol use during pregnancy	0	0	n/a
Maternal hepatitis C, *n* (%)	5 (21.7)	0	n/a
Maternal college degree, *n* (%)	1 (4.3)	15 (51.7)	0.013
Maternal methadone, *n* (%)	4 (17.4)	n/a	n/a
Maternal buprenorphine, *n* (%)	18 (78.3)	n/a	n/a
Maternal illicit opioids (e.g., heroin and/or fentanyl), *n* (%)	8 (34.8)	n/a	n/a
Other maternal non-opioid illicit drug use, *n* (%)	6 (26.1)	0	n/a
Neonatal abstinence syndrome requiring opioid treatment, *n* (%)	5 (21.7)	n/a	n/a
Length of infant Hospital stay, mean days (SD)	11.78 (11.77)	2.03 (0.82)	<0.001

*Unpaired two-sided t-test was used to compare continuous variables and Chi square test was used to compare categorical variables*.

### CAP Analysis

**Figure 1:**
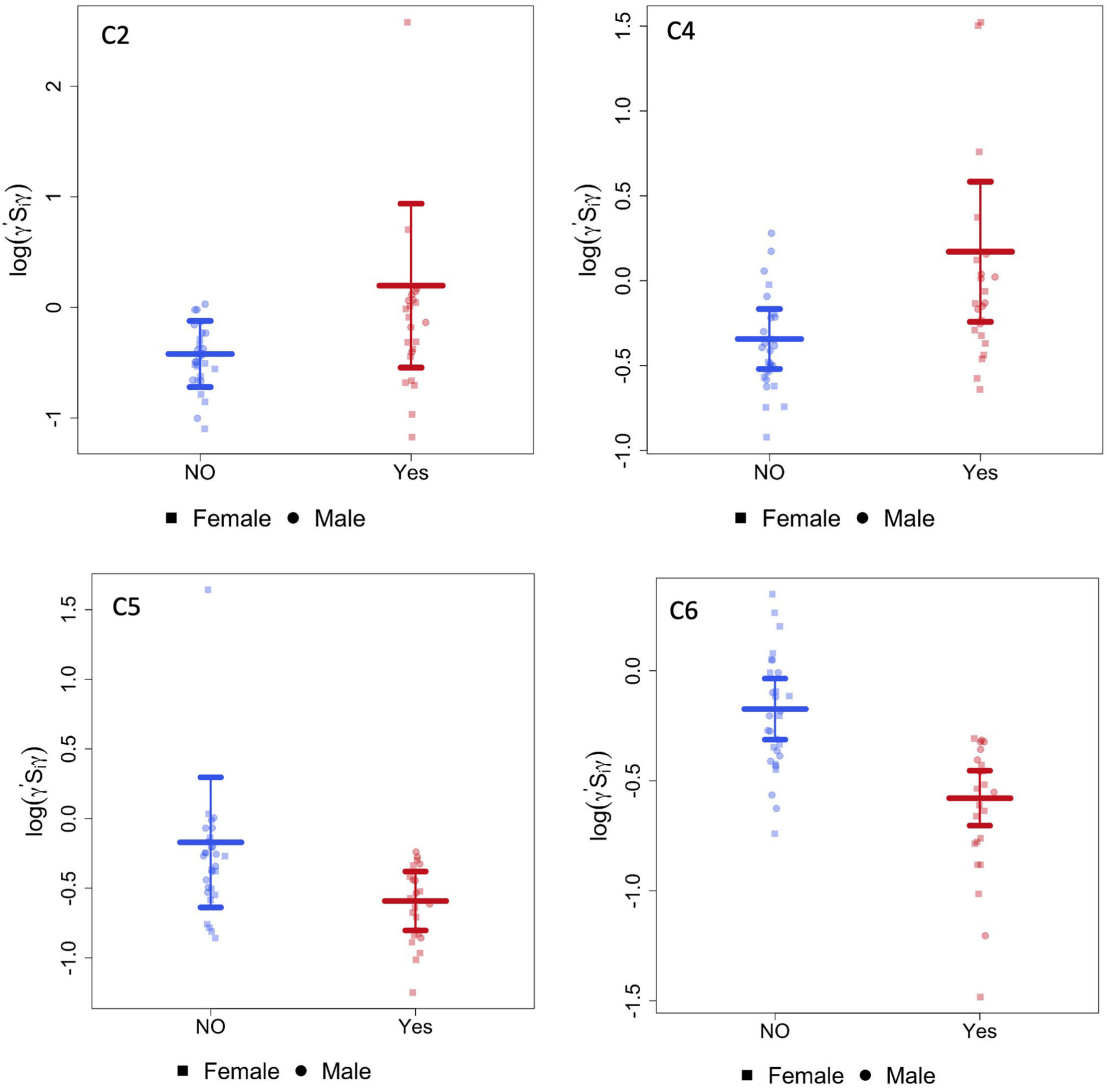
CAP networks. Graphs of gestational age and sex adjusted intra-network connectivity in infants with POE (labeled “yes”) and control infants (labeled “NO”) for the CAP networks (C2,C4, C5, and C6) that were significantly different between the two groups after correcting for multiple network comparisons as well as for gestational age and sex.

The CAP method identified six brain subnetworks and four of them (denoted by C2, C4, C5, and C6) indicated a significant difference in functional connectivity between infants with opioid exposure and controls. Table 2 and Figure 1 present the estimated model coefficients and the 95% confidence interval in these four subnetworks. Two of these networks, C2 and C4 had a positive coefficient suggesting that the within-network connectivity among opioid-exposed infants is stronger than the controls. C2 consists of regions of inferior temporal gyrus (ITG, left and right), superior temporal pole (TPO, right), inferior frontal gyrus (IFG) triangularis (left), IFG opercularis (right), inferior occipital gyrus (IOG, left and right), dorsal superior frontal gyrus (SFG, left and right), middle frontal gyrus (MFG, right), precentral gyrus (left), middle and inferior orbitofrontal cortex (ORB, right), insula (right), superior parietal gyrus (SPG, left), and inferior parietal lobule (IPL, right). C4 includes regions of superior ORB (right), middle ORB (left), IFG triangularis (left and right), middle cingulate gyrus (MCG, left), hippocampus (left), middle and superior occipital gyrus (MOG and SOG, left), postcentral gyrus (right), angular gyrus (ANG, right), paracentral lobule (PCL, right), and ITG (right).

**Table 2:** Estimated coefficients of the CAP regression model.

	**CAP 2**	**CAP 4**	**CAP 5**	**CAP 6**
Opioid	0.62 (0.07, 1.16)	0.51 (0.17, 0.85)	−0.42 (−0.74, −0.10)	−0.40 (−0.55, −0.26)
Male	−0.38 (−0.75, −0.01)	−0.51 (−0.76, −0.25)	−0.44 (−0.82, −0.05)	−0.35 (−0.50, −0.20)
Gestational age	−0.18 (−0.57, 0.22)	0.03 (−0.19, 0.26)	0.17 (0.05, 0.29)	0.23 (0.14, 0.31)

*Estimated model coefficient and 95% confidence interval from 500 bootstrap samples in the CAP regression model for the subnetworks with a significant difference between opioid exposed infants and controls. The presence of prenatal opioid exposure was considered the variable of interest and the analyses were adjusted for the effects of sex and gestational age*.

Two of the CAP networks, namely C5 and C6 had a negative coefficient indicating lower within-network connectivity among opioid-exposed infants compared to controls. C5 includes regions of dorsal SFG (left), MFG (left and right), inferior ORB (right), medial SFG (left and right), anterior cingulate gyrus (ACG, left and right), lingual gyrus (LING, right), fusiform gyrus (FFG, left), SPG (right), superior temporal gyrus (STG, left), and ITG (right). Regions in C6 network include medial ORB (right), inferior ORB (left), olfactory (OLF, left), medial SFG (left and right), cuneus (CUN, left), LING (left), MOG (right), precuneus (PCUN, right), and ITG (right).

CAP networks C5 and C6 were significantly associated with presence of maternal psychological factors (Table 3) with negative coefficients indicating intra-network hypoconnectivity in the presence of maternal psychological factors. We did not identify significant correlations of the CAP networks with presence of maternal smoking, however, when smoking was introduced into the model, the effect size of CAP 2 network increased and the rest (CAP 4, CAP 5, and CAP 6) decreased (Supplementary Table 1). Similarly, we did not identify significant correlations of the CAP networks with presence of maternal polysubstance exposure.

**Table 3:** Association estimates of the maternal risk factors and CAP network components.

**Maternal covariates**	**CAP C2**		**CAP C4**		**CAP C5**		**CAP C6**	
	**β (95% CI)**	***p*** **-value**	**β (95% CI)**	***p*** **-value**	**β (95% CI)**	***p*** **-value**	**β (95% CI)**	***p*** **-value**
Maternal psychological factors	0.3 (−0.06; 0.66)	0.3	0.13 (−0.2; 0.46)	1	−0.39 (−0.66; −0.12)	0.018	−0.46 (−0.73; −0.2)	0.0027
Maternal polysubstance use	0.29 (−0.38; 0.95)	1	−0.34 (−0.91; 0.23)	0.66	0.13 (−0.13; 0.34)	0.93	0.21 (−0.11; 0.54)	0.54
Maternal smoking	−0.21 (−0.91; 0.48)	1	0.25 (−0.36; 0.85)	1	0.06 (−0.23; 0.34)	1	−0.02 (−0.37; 0.33)	1

*Association estimates of the maternal risk factors and the CAP network components that were significantly different between infants with prenatal opioid exposure and control infants. For each network, Bonferroni method was used for correcting for multiple comparisons. Positive estimates indicate higher intra-network functional connectivity in the presence of that maternal covariate, and negative estimates indicate lower intra-network functional connectivity in the presence of that maternal covariate*.

### Graph Network Analysis

**Figure 2:**
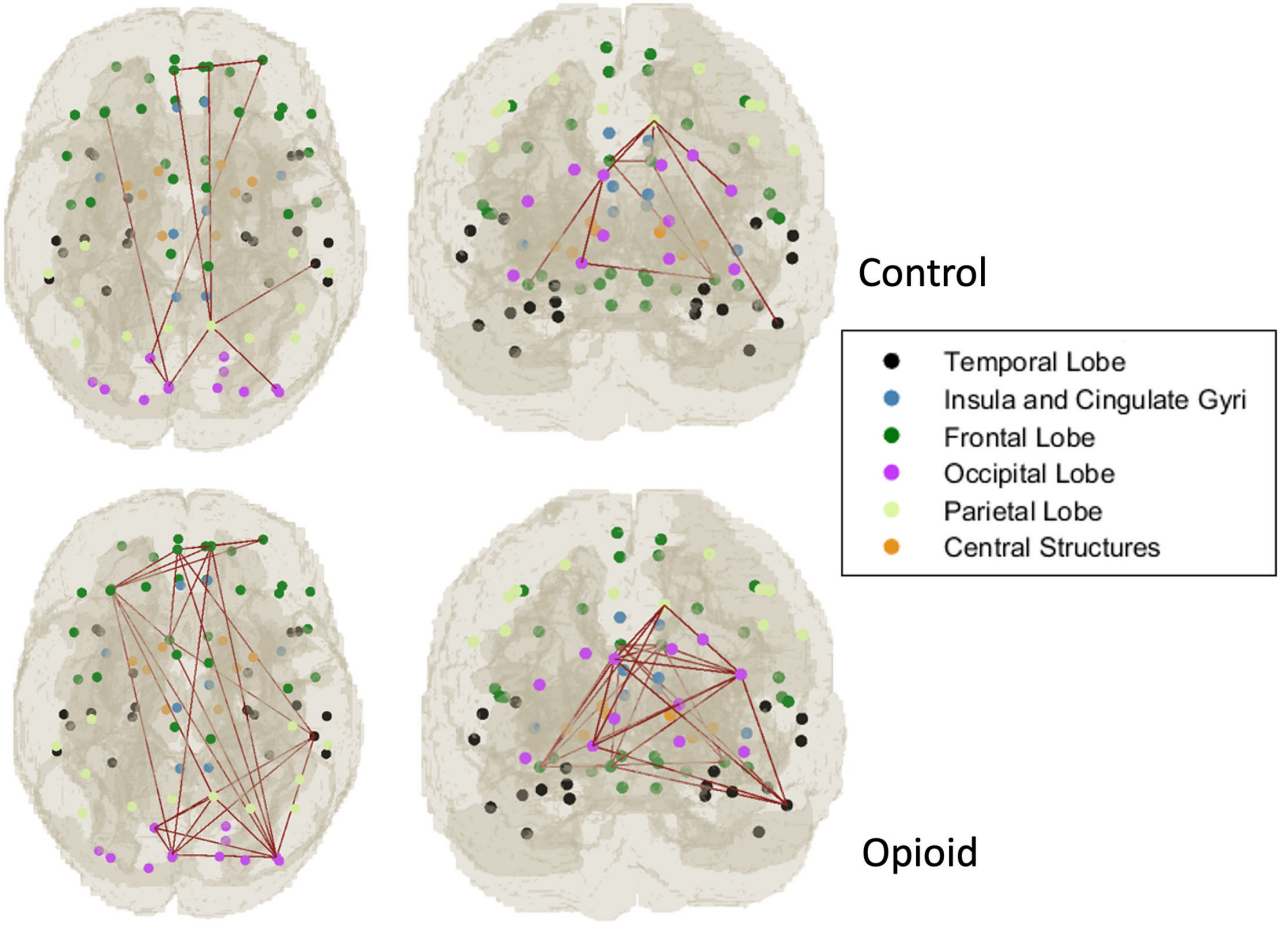
Graph network modeling representation. Representative example of single subject graph network modeling for CAP C6 network in a control infant (top row) and infant with POE (bottom row). The dots refer to the center of the atlas region of interest and the connecting lines represent the thresholded graph network connections in these subjects.

For the CAP network components that were significantly different between infants with POE and control infants, graph network analysis revealed the subnetwork from CAP component 6 to have significant group difference in average flow coefficient, clustering coefficient and transitivity after accounting for sex and gestational age and correcting for intra-network analyses (Table 4, Figure 2). There was no significant difference in other network level measures such as assortativity, density, global efficiency, and modularity.

**Table 4:** Significance of difference in graph network-level topological measures.

**Network-level measures**	**CAP C2**	**CAP C4**	**CAP C5**	**CAP C6**
Assortativity	0.96	0.98	0.77	0.67
Average flow coefficient	0.96	0.98	0.96	0.03
Density	0.96	0.98	0.96	0.24
Global efficiency	0.96	0.98	0.96	0.4
Modularity	0.96	0.98	0.96	0.67
Clustering coefficient	0.96	0.98	0.77	0.03
Transitivity	0.96	0.98	0.96	0.03

*Significance of difference in graph network-level topological measures between infants with prenatal opioid exposure and control infant groups on the CAP networks that were significantly different between the POE and control groups. For each network, FDR correction for multiple comparisons was performed*.

## Discussion

This study comparing prenatal opioid exposed infants with healthy infants without prenatal opioid exposure demonstrates significant alterations in global brain network connectivity in infants with POE, with some of these networks also showing alterations in graph theory metrics. Most importantly, our study identified that some of these global brain network connectivity alterations were associated with maternal psychological factors, a common co-morbidity in pregnant women with opioid use disorder. This suggests a role of the interplay between maternal opioid use and psychological disorders on the developing infant brain.

Brain resting state functional networks exist in infants and have recently been shown to be altered in the presence of prenatal exposure to substances (27, 28, 57). However, prenatal substance exposure has also shown to be associated with alterations in multiple other brain regions, both intra-network and inter-network, outside of these canonical resting state functional networks (57). Our study focused on a network-free model of global brain functional connectivity, rather than predefined networks. In our study, the identified CAP networks associated with POE spanned all lobes of the brain including the frontal, temporal, occipital, parietal and insula, suggesting a potential global influence of prenatal substance exposure on the developing brain. Many of the brain regions that comprised the CAP networks have been described as components of several networks including salience, executive function, rewards, dorsal and ventral attention, somatosensory, language, and visual networks. Two of the CAP networks in our study showed higher intra-network connectivity in infants with POE compared to control infants, while two of the networks showed lower intra-network connectivity. Similar findings of network hyper- and hypo-connectivity have been described in infants with opioid and other prenatal substance exposure (58). Functional MRI studies in adults with drug addiction reveal intra-network hyperconnectivity in the reward and salience networks and hypoconnectivity in the executive function network, the medial frontal regions, anterior cingulate gyrus and temporal regions (59, 60). We postulate that the CAP network alterations in infants with POE observed in our study, are a combination of hyperconnectivity and hypoconnectivity within and between these known resting state functional networks.

There have been a few recent small cohort studies showing alterations in brain resting state functional connectivity in infants with prenatal opioid exposure (27, 28). To date, ours is the largest cohort study focused on infants with POE. While Merhar et al. also studied global brain networks in infants with POE, and their results suggest correlations of brain connectivity with Finnegan score, we did not find any such correlation in our study, which is likely due to the differences in methodology, as we used the CAP method for brain network assessment after controlling for gestational age and sex (27). Additionally, we also identified alterations in infant brain resting state functional network connectivity associated with maternal psychological factors. Specifically, presence of maternal psychological factors showed intra-network hypoconnectivity in infants with POE in CAP C5 and C6, suggesting that maternal psychological factors should be considered when assessing for opioid related effects on the developing brain.

Imaging studies have also identified alterations in infant brain development in the setting of antenatal maternal depression, with these brain functional alterations shown to be associated with short term infant behavioral outcomes. Alterations in amygdala functional connections to several regions including the prefrontal cortex are identified in infants born to mothers with prenatal depression compared to control healthy infants (36, 37). Infant brain functional network hub distribution was also shown to be altered in those exposed to prenatal maternal depression, and these brain network alterations were predictive of 6 month infant temperament variance, highlighting the clinical relevance of these altered neonatal brain functional organization (61). Maternal depression and anxiety have also been associated with altered white matter microstructure in infants, especially decreased neuritic density and increased mean, radial and axial diffusivity in specific brain regions (62). SSRIs used to treat depression are also shown to be associated with the relationship between infant brain functional connectivity and early infant behavior (57); however, none of the women in our study were on SSRIs during pregnancy. We did not identify any significant effects of polysubstance use on altered infant brain functional connectivity in our cohort of prenatal opioid exposed infants, which may partly be explained by the differential “signatures” of altered infant brain functional connectivity of different prenatal substance exposures (57, 58).

Since the fetal period and infancy are a period of rapid and dynamic brain development, there are likely to be variable effects of POE on the maturing brain during this period, and hence variable effects on long term cognition and behavioral outcomes, which may also be modified by the presence or absence of opioid maintenance therapy, exposure to other substances or nicotine, maternal psychological factors and various demographic factors such as socioeconomic status, maternal education, and employment. Understanding this complex interplay of prenatal and postnatal factors in the setting of POE and maternal mental health on brain development and their effects on long term outcomes is key to developing optimal intervention strategies in this population. Visual and visual-motor problems are suggested to be more common in children with POE (63), but there is no convincing evidence for long-term cognitive impairment. While a few studies indicate increased risks for educational delay, lower IQ, and poor language performance in children with POE, other studies show no significant differences in cognitive outcomes (63–67). There is, however, greater evidence to suggest an increased risk of poor long-term behavioral outcomes in children with POE, including difficulties with executive functioning (67), attention deficit hyperactivity disorder (ADHD) (68, 69) and autism spectrum disorders (ASD) (70, 71). Children with POE and a history of NOWS are shown to be more likely to have poor neurodevelopmental consequences including lower IQ, poor educational testing performance, lower attention, meet disability criteria, and require additional classroom therapies and services when compared to children with POE who did not develop NOWS, or healthy controls (18, 71–74).

Our study has a few limitations. Opioid use in pregnant women is a complex issue, often associated with several other socioenvironmental comorbidities, as well as maternal confounders including polysubstance use, smoking and psychological factors, which may independently influence infant brain development. Although we were able to show the primary differences between the opioid-exposed and healthy infant brain connectivity, this study was not powered to study all maternal socioenvironmental factors such as maternal education level. We showed that maternal psychological factors correlated significantly with infant brain global brain network connectivity. However, many mothers of the POE group also had polysubstance use and smoking while none of the controls reported substance use or smoking. Although we did not find a significant correlation of smoking and polysubstance use with the altered infant brain networks in our study, in the absence of a control group with these covariates, it remains a challenge to assign the cause of the findings solely to opioid use. Moreover, the long-term clinical significance of the observed changes is currently unknown and could be evaluated by large scale longitudinal studies, such as the NIH HEALthy BCD study, which should also address the effects other confounding maternal and environmental factors.

In conclusion, our study showed significant alterations in global network level resting state functional connectivity in infants with POE, compared to healthy infants without prenatal opioid exposure, with altered graph network metrics within these regions. Additionally, these alterations in global brain network connectivity were significantly associated with the presence of maternal psychological factors. Since alterations in global brain connectivity may play a role in developmental outcomes, our study suggests the need for large prospective longitudinal studies to understand the prognostic ability of these early functional brain network alterations, and the benefits of early intervention on childhood long term development.

## Data Availability Statement

The original contributions presented in the study are included in the article/Supplementary Material, further inquiries can be directed to the corresponding author/s.

## Ethics Statement

The studies involving human participants were reviewed and approved by Indiana University Institutional Review Board. Written informed consent to participate in this study was provided by the participants' legal guardian/next of kin.

## Author Contributions

RR, YZ, and JY: conceptualization. RR, YZ, DH, GS, JY, BH, NS, RV, and SS: methodology and review of final manuscript. RR, YZ, NS, RV, JY, and BH: formal analysis and investigation. RR, RV, and SS: writing—original draft preparation. RR, YZ, DH, JY, BH, RV, and SS: writing—review and editing. RR and SS: funding acquisition. RR, YZ, JY, and SS: resources. RR, JY, and SS: supervision. All authors contributed to the article and approved the submitted version.

## Funding

RR was supported by the American Roentgen Ray Scholarship Award 2018 and Radiological Society of North America Seed Grant 2018. SS and RR were supported by the Eunice Kennedy Shriver National Institute of Child Health & Human Development of the National Institutes of Health under Award, R01HD096800 (PI: SS).

## Author Disclaimer

The content is solely the responsibility of the authors and does not necessarily represent the official views of the National Institutes of Health.

## Conflict of Interest

The authors declare that the research was conducted in the absence of any commercial or financial relationships that could be construed as a potential conflict of interest.

## Publisher's Note

All claims expressed in this article are solely those of the authors and do not necessarily represent those of their affiliated organizations, or those of the publisher, the editors and the reviewers. Any product that may be evaluated in this article, or claim that may be made by its manufacturer, is not guaranteed or endorsed by the publisher.

## Abbreviations

R: right
L: left
POE: prenatal opioid exposure
NOWS: neonatal opioid withdrawal syndrome
NAS: neonatal abstinence syndrome
ROI: region of interest
MRI: magnetic resonance imaging
rs-fMRI: resting state functional magnetic resonance imaging
FD: framewise displacement
DVARS: temporal Derivative of timecourses root-mean-squared VARiance over voxels
ADHD: Attention deficit hyperactivity disorder.
