# Supplementary material for: Global Brain Functional Network Connectivity in Infants With Prenatal Opioid Exposure
Source: Front Pediatr. 2022 Mar 14;10:847037. doi: 10.3389/fped.2022.847037 (PMC8964084; doi:10.3389/fped.2022.847037)
Supplement: Supplementary file 1 [file Table_1.DOCX]

Table S1. Estimated coefficients of the CAP regression model after including (a) smoking or (b) maternal psychological factors

1. Smoking

|  | CAP 2 | CAP 4 | CAP 5 | CAP 6 |
| --- | --- | --- | --- | --- |
| Opioid | 0.97 (0.12, 2.00) | 0.35 (0.08, 0.60) | -0.35 (-0.53, -0.19) | -0.36 (-0.48, -0.22) |
| Male | -0.33 (-0.59, -0.07) | -0.47 (-0.64, -0.31) | -0.42 (-0.76, -0.15) | -0.37 (-0.48, -0.26) |
| Gestational Age | -0.08 (-0.19, 0.02) | 0.07 (-0.04, 0.18) | 0.16 (0.10, 0.22) | 0.21 (0.17, 0.25) |
| Maternal smoking | -0.67 (-1.71, 0.31) | 0.30 (-0.13, 0.73) | 0.01 (-0.14, 0.15) | -0.05 (-0.22, 0.12) |

1. Maternal psychological factors

|  | CAP 2 | CAP 4 | CAP 5 | CAP 6 |
| --- | --- | --- | --- | --- |
| Opioid | 0.45 (0.26, 0.65) | 0.53 (0.32, 0.74) | -0.35 (-0.58, -0.15) | -0.38 (-0.47, -0.30) |
| Male | -0.41 (-0.66, -0.13) | -0.49 (-0.67, -0.30) | -0.38 (-0.65, -0.12) | -0.35 (-0.46, -0.26) |
| Gestational Age | -0.07 (-0.17, 0.03) | 0.07 (-0.05, 0.19) | 0.14 (0.08, 0.20) | 0.20 (0.17, 0.23) |
| Maternal psychological factors | 0.40 (-0.21, 0.97) | -0.02 (-0.36, 0.29) | -0.14 (-0.28, -0.01) | -0.12 (-0.24, -0.01) |

Note: Estimated model coefficient and 95% confidence interval from 500 bootstrap samples in the CAP regression model for the subnetworks with a significant difference between opioid exposed infants and controls. Intra-network connectivity is the dependent variable. The primary independent variable of interest is the prenatal opioid exposure. We adjusted for rest of the independent variables - sex, gestational age, and (a) maternal smoking/(b) maternal psychological factors by including them in the regression model. The table provides the estimated coefficient of these variables.
